# Lights and shadows of cardiac magnetic resonance imaging in acute myocarditis

**DOI:** 10.1007/s13244-015-0444-7

**Published:** 2015-11-10

**Authors:** Antonio Esposito, Marco Francone, Riccardo Faletti, Maurizio Centonze, Filippo Cademartiri, Iacopo Carbone, Roberto De Rosa, Ernesto Di Cesare, Ludovico La Grutta, Guido Ligabue, Luigi Lovato, Erica Maffei, Riccardo Marano, Massimo Midiri, Gianluca Pontone, Luigi Natale, Francesco De Cobelli, From the Working Group of the Italian College of Cardiac Radiology by SIRM

**Affiliations:** IRCCS Ospedale San Raffaele, Università Vita-Salute San Raffaele, Milan, Italy; Department of Radiological, Oncological and Pathological Sciences, Sapienza University of Rome, V.le Regina Elena 324, 00161 Rome, Italy; Radiologia Università Torino, Turin, Italy; Radiologia OC S. Chiara Trento, Trento, Italy; Department of Radiology, Montreal Heart Institute, Universitè de Montreal, Montreal, Canada; ASL Napoli 1, Naples, Italy; Department of Biotechnological and Applied Clinical Sciences, University of L’Aquila, L’Aquila, Italy; Palermo University, Palermo, Italy; Azienda Ospedaliera-Universitaria Policlinico di Modena, Modena, Italy; Policlinico S. Orsola Bologna, Bologna, Italy; Università Cattolica Roma, Rome, Italy; IRCCS Centro Cardiologico Monzino, Milan, Italy

**Keywords:** Acute myocarditis, Cardiac magnetic resonance, T1 mapping, Lake Louise criteria

## Abstract

**Abstract:**

Cardiac magnetic resonance (CMR) is considered a primary tool for the diagnosis of acute myocarditis, due to its unique potential for non-invasive identification of the various hallmarks of the inflammatory response, with relevant impact on patient management and prognosis. Nonetheless, a marked variation in sensitivity and negative predictive value has been reported in the literature, reflecting the intrinsic drawbacks of current diagnostic criteria, which are based mainly on the use of conventional CMR pulse sequences. As a consequence, a negative exam cannot reliably exclude the diagnosis, especially in patients who do not present an infarct-like onset of disease. The introduction of new-generation mapping techniques further widened CMR potentials, allowing quantification of tissue changes and opening new avenues for non-invasive workup of patients with inflammatory myocardial disease.

***Main messages*:**

• *CMR sensitivity varies in AM, reflecting its clinical polymorphism and the intrinsic drawbacks of LLc.*

• *Semiquantitative approaches such as EGEr or T2 ratio have limited accuracy in diffuse disease forms.*

• *T1 mapping allows objective quantification of inflammation, with no need to normalize measurements.*

• *A revised protocol including T2-STIR, T1 mapping and LGE could be hypothesized to improve sensitivity.*

## Introduction

Acute myocarditis (AM) is a “multifaceted disease” characterized by a large variety of acute manifestations, potentially followed by unpredictable sequelae ranging from dilated cardiomyopathy to recurrent arrhythmias [1, 2]. Although relatively common in clinical practice [2, 3], AM is underdiagnosed in the community due to the low sensitivity of conventional diagnostic tools such as ECG, cardiac biomarkers, and viral serology [1]. The use of endomyocardial biopsy (EMB) [4] is controversial, given the lack of standardized protocols and the high prevalence of sampling errors related to the common patchy distribution of myocardial inflammation [4, 5].

**Fig. 1:**
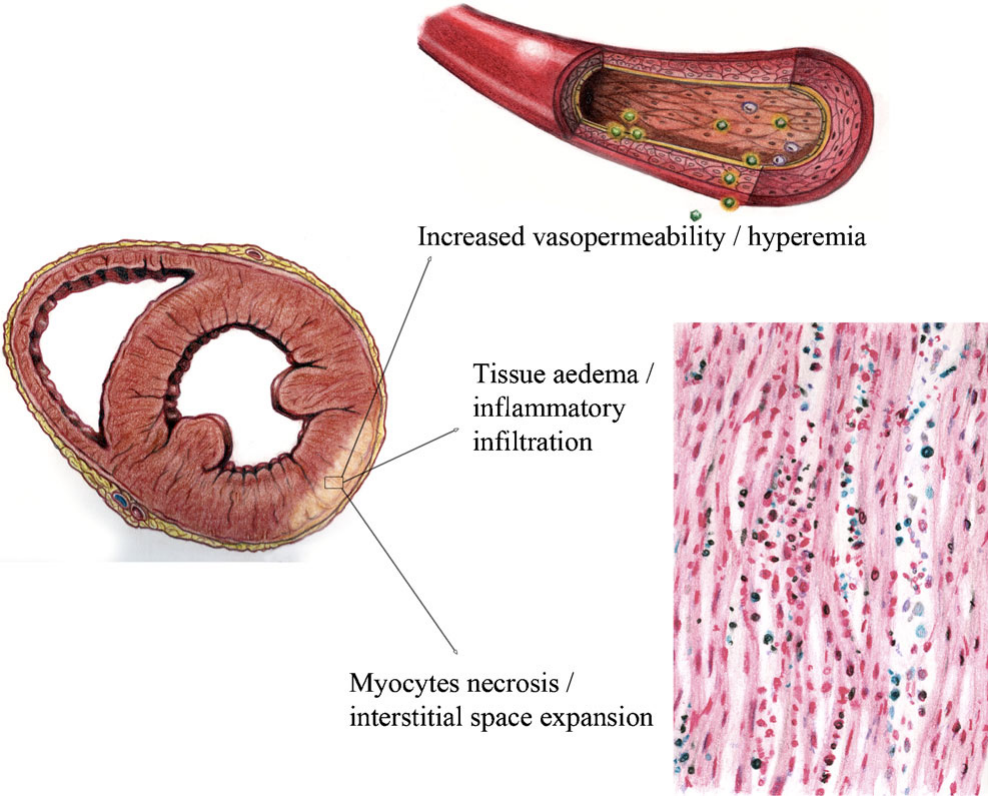
Freehand drawing schematically representing tissue targets of CMR in acute myocarditis. A typical inferior-lateral left ventricular involvement with subepicardial spread is depicted. In most cases, the process derives from a viral infection that induces myocardial necrosis (or apoptosis) and triggers an immunoreactive response, with subsequent vasoactive phenomena and tissue oedema (freehand drawing by Bettina Conti, MD, Sapienza University of Rome)

In this complex scenario, cardiac magnetic resonance (CMR) was immediately perceived as a potentially “revolutionary” non-invasive tool [6–9], with a unique capacity to characterize the typical hallmarks of myocardial inflammation (Fig. 1). Ghelani et al., for example, reported a retrospective study among a population of 514 patients demonstrating a fivefold increase (from 5.2 to 28.1 %) in the utilization of CMR in AM over a 10-year period from 2006 to 2015, with a significant parallel decline in the rate of EMB [10].

Current diagnostic criteria (Lake Louise criteria; LLc) are based on the application of a recommended standardized protocol that aims to identify typical signs of the inflammatory cascade, which consist of oedema, regional hyperemia, and cellular death [11]. These criteria have been extensively validated in the literature, and despite suboptimal diagnostic accuracy of about 78 %, are commonly applied in clinical routine [12, 13]. Promising novel T1/T2 mapping techniques have recently been introduced that provide quantitative measurements of tissue changes, minimizing subjectivity and errors common in conventional threshold-based post-processing methods [12–14].

This document analyses lights and shadows of CMR imaging in the diagnostic and prognostic assessment of AM and evaluates the improvement provided by the introduction of T1/T2 mapping techniques into clinical routine.

### Diagnostic criteria in the pre-mapping era: lights and shadows

The spectrum of currently available sequences for CMR study of myocarditis is summarized in Table 1.

**Table 1:** Information provided by various CMR techniques applied in the evaluation of acute myocarditis, with main imaging features

CMR technique	Information provided	Imaging features
Cine-SSFP	Regional and global biventricular function, ventricular mass, and parietal wall thickness	- Normal or mildly dilated left or biventricular cavities
		- Ejection fraction depending on clinical presentation, usually mildly depressed (45–50 %)
		- Parietal wall thickness normal or slightly increased (>10 mm)
		- Pericardial effusion in 30–50 % of cases
T2w-STIR	Increased myocardial free water content	- Subepicardial or patchy areas of high signal intensity following LGE distribution
		- Global hyperintensity compared to skeletal muscle (T2 ratio > 1.9 according to LLc)
Pre- and post-Gd T1w FSE	Myocardial hyperemia and expansion of extracellular compartment	- Sequences frequently affected by severe artefacts
		- Myocardial hyper-enhancement compared to skeletal muscle (EGEr > 4 according to LLc)
Delayed enhancement	Myocardial necrosis, scars	- No enhancement
		- Focal subepicardial enhancement typically involving inferolateral LV wall
		- Patchy or longitudinal striae of mid-wall enhancement
Native T1 mapping	Pixel-by-pixel assessment of T1-rt revealing myocardial changes, first of all oedema	- T1-rt prolongation: proposed cut-off > 990 ms (59)
Pre- and post-Gd T1 mapping	ECV expansion due to enhanced diffusion of free water and cardiomyocyte apoptosis	- ECV increase: proposed cut-off ≥ 27 %; still few published data (34)
T2 mapping	Pixel-by-pixel assessment of T2-rt revealing myocardial oedema	- T2-rt prolongation; still few published data (64)

*CMR* cardiovascular magnetic resonance, *SSFP* steady-state free precession, *T2w-STIR* T2-weighted short-tau inversion recovery, *FSE* fast spin echo, *Gd* gadolinium, *T1-rt* T1 relaxation time, *ECV* extracellular volume, *T2-rt* T2 relaxation time, *LGE* late gadolinium enhancement, *LLc* Lake Louise criteria, *EGEr* early gadolinium enhancement ratio, *LV* left ventricle

According to the LLc [11], CMR diagnosis of AM can be established in the presence of at least two of the following three features:

- myocardial oedema detected by T2-weighted techniques;
- myocardial hyperemia detected by early gadolinium enhancement (EGE) techniques;
- myocardial damage with non-ischemic pattern detected by late gadolinium enhancement (LGE) techniques.

**Fig. 2:**
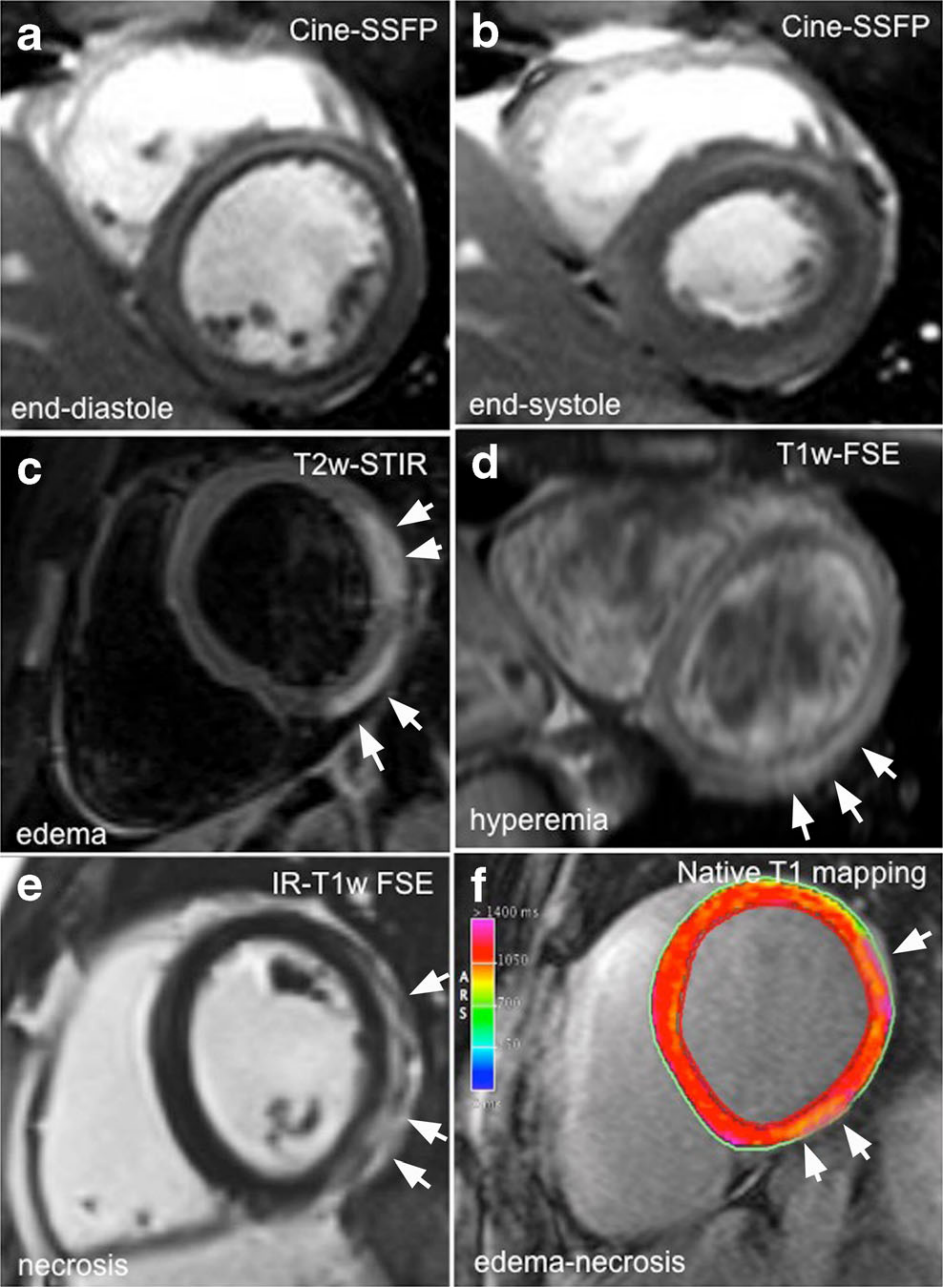
Comprehensive CMR evaluation in a 27-year-old man with acute myocarditis following an upper respiratory infection. At admission, patient presented with acute chest pain, abnormal ST segment elevation in the inferolateral leads, and mildly elevated troponin; left ventricular global function was preserved (**a**, **b**; ejection fraction 56 %). Typical hallmarks of active myocardial inflammation are portrayed, consisting of meso/subepicardial striae of high signal intensity on both T2-STIR and LGE images (**c**–**e**), combined with a positive EGEr (**d**; 7.6). Corresponding native T1 parametric map calculated using a modified Look-Locker inversion recovery (MOLLI) sequence with a 1.5 T magnet (MAGNETOM Avanto; Siemens Healthcare, Erlangen, Germany) shows increased T1 values in the same location (1211 ± 16 ms; normal reference value mean 1027 ± 61 ms; **f**)

These three criteria are considered in addition to steady-state free precession (SSFP) sequences for the assessment of biventricular volumes and global/regional function (Fig. 2).

1. OEDEMA and INFLAMMATORY INFILTRATION:
  Interstitial or intracellular accumulation of water, together with serological biochemical markers, represents the most reliable sign of active inflammation [15, 16].
  Currently, the most widely used and validated technique for “oedema-weighted” imaging is based on the application of T2-weighted short-tau inversion recovery sequences (T2w-STIR) [15, 17, 18]. The STIR technique includes a third 180° inversion pulse integrated into the classical double-inversion recovery black blood turbo spin echo (TSE) pulse sequence. The first and second inversion pulses create a black blood effect; the third inversion pulse leads to the suppression of the signal from fatty tissue and from other stationary tissue with a T1 relaxation time of approximately 200–250 ms. Another important effect of the third pulse enabling the detection of oedema is the effect of the inversion of the long T1 on final signal intensity, so the T1 lengthening related to oedema contributes to the increased signal of oedematous tissue, together with T2 lengthening [19].
  Myocardial oedema in AM often proceeds from a focal meso- and subepicardial distribution, which is observed in only about 30 % of patients, to a global pattern of ventricular involvement.
  Current diagnostic criterion:
    **Definition**:
    T2w-STIR sequences must be performed using the same prescription of SSFP images in short-axis views and in three long-axis planes (two-, three-, and four-chamber views). However, a body coil is recommended to avoid signal heterogeneity as compared to SSFP.
    The presence of tissue oedema may be detected visually or, as suggested in the LLc, using a semiquantitative method. Regional oedema is defined as region of at least 10 adjacent pixels with signal intensity more than 2 standard deviations above the mean value of normal tissue, evident in two orthogonal planes [11].
    Diffuse oedema is quantified as the ratio of myocardial to skeletal muscle (T2 ratio) by drawing two distinct regions of interest in the same slice; a ratio ≥ 1.9 is considered positive and reflective of global myocardial oedema [16]. An example of posterolateral left ventricle oedema is showed in Fig. 1.
    **Limitations**:
    T2w-STIR images usually allow reliable visual identification of oedema in patients with a focal pattern of myocardial involvement, although the limited contrast-to-noise ratio (CNR) may impede the detection of subtle tissue changes in a significant minority of patients.
    Other weaknesses may prevent the interpretation of T2w-STIR images under certain conditions. First, signal heterogeneity related to coil sensitivity profiles can produce both false-negative and false-positive results, and thus the use of reliable surface coil intensity correction filters or body coils is of pivotal importance. Second, the dark blood preparation pulse may introduce regional signal loss in the LV wall, especially in the posterior and posterolateral wall, caused by through-plane cardiac motion. Third, incomplete dark blood preparation may lead to bright rim blood artefacts along the endocardium, especially in patients with impaired ventricular function [20].
    Another limitation is the qualitative nature of T2w-STIR images, which implies the need for reference tissue for interpretation. In the case of suspected diffuse oedema, the reference tissue is the skeletal muscle, although a concomitant signal change within skeletal muscles, such as that in patients with coexisting myositis, may adversely affect diagnostic performance [21, 22].
    Finally, a cut-off value of 1.9 was defined using clinical criteria (symptoms, ECG and serologic evidence of myocardial injury, and angiographic exclusion of coronary artery disease) as a reference standard for establishing a diagnosis, without histological validation [16].
2. HYPEREMIA:
  The identification of myocardial hyperemia is the second step of the diagnostic workup, according to the LLc. Endothelial dysfunction and increased vasopermeability have been considered the pathophysiological mechanisms for abnormal diffusion of gadolinium from the vascular compartment to the interstitial space. The tissue hyperemia is evaluated with a technique commonly defined as EGE [11, 16].
  Current diagnostic criterion:
    **Definition**:
    The technique used to assess myocardial hyperemia in the context of LLc was originally proposed by Abdel-Aty et al. in 2005. A free-breathing black blood fast spin echo (FSE) T1w sequence with an acquisition time of 3–4 min is acquired in four identical axial slices, both before and after (without any change in the parameters in between) intravascular injection of 0.1 mmol of gadopentetate dimeglumine (Gd-DTPA) [11].
    Sequence parameters should be adjusted to maximize T1 weighting; in particular, an echo train length of less than 4 is desirable. According to LLc, diffuse hyperemia can be detected by calculating the early gadolinium enhancement ratio (EGEr): endocardial and epicardial contours should be traced on both pre- and post-contrast images, and a reference region of interest (ROI) should be placed on the skeletal muscle located within the same slice. The myocardial enhancement should be normalized on the skeletal muscle enhancement, and an EGEr ≥ 4 is considered positive for myocardial inflammation [11].
    *Limitations*:
    EGE imaging is generally considered the least robust of the three components of the LLc, as FSE sequences originally proposed [11] are limited by inconsistent image quality in many patients [21]. In particular, this sequence is highly prone to severe respiratory artefacts caused by free breathing during acquisition.
    Second, the acquisition of only 3–4 subsequent slices limits anatomic coverage of the left ventricle, impeding a panoramic assessment of the myocardial wall.
    Third, the cut-off value is validated with one standard-relaxivity contrast medium (Gd-DTPA) and may be not acceptable if higher relaxivity agents are used (e.g., Gd-BOPTA and gadobutrol). Therefore, a redefinition of thresholds is needed for agents with higher relaxivity, as has already been reported in a similar model of breast MRI [23].
    Fourth, signal normalization may be hampered by coexisting skeletal muscles disease. Hence, in patients with an increase in skeletal muscle signal intensity ≥ 20 %, as well as in patients with a recent history of muscular pain, an increase of > 45 % in absolute myocardial signal intensity between pre- and post-gadolinium images is suggested as a threshold consistent with myocarditis, rather than normalized EGEr [11].
    An interesting alternative method for fast assessment of myocardial hyperemia in AM was recently reported, which relies on SSFP sequences acquired soon after contrast administration (ceSSFP) [24]. This approach overcomes most of the drawbacks of original FSE sequences and seems to be effective for the identification of areas of regional hyperemia, although it is not yet validated in patients with EMB-proven AM. However, the ceSSFP technique has not yet been implemented in the assessment of diffuse changes involving the entire LV wall, and this is its main limitation.
3. MYOCYTE DEATH and FIBROSIS
  Myocyte necrosis with extracellular space expansion followed by replacement fibrosis occurs in AM as a consequence of either direct viral cellular damage or cell-mediated immunological injury. Both tissue changes lead to an increase in delayed gadolinium accumulation, with subsequent positive LGE. Typical disease patterns are characterized by mid-wall or subepicardial LGE distribution, with patchy or linear areas of high signal intensity [25]. The inferolateral mid-basal wall and the septum are the more involved segments [26]. This pattern is clearly different from the ischemic pattern, the latter characterized by subendocardial or transmural LGE, fitting the segmental distribution of coronary arteries, with possible associated microvascular obstruction.
  Several sequences are available today for the assessment of LGE, including the two-dimensional (2D) and three-dimensional (3D) inversion recovery gradient echo sequences (IR-GRE), the 2D and 3D inversion recovery SSFP sequences (IR-SSFP), and the phase-sensitive inversion recovery sequences (PSIR-GRE or PSIR-SSFP) that eliminate the need for a precise null time for normal myocardium. This sequence uses phase-sensitive detection to remove the background phase while preserving the signal of the desired magnetization during IR [27].
  Figure 2 (Panel E) shows a typical subepicardial LGE of the lateral wall.
  Current diagnostic criterion:
    **Definition**:
    LGE is considered positive if at least one focal area of high signal intensity with non-ischemic pattern of distribution is outlined in at least two orthogonal planes [11].
    Suggested LLc protocol recommends the application of conventional segmented two-dimensional breath-hold IR-GRE pulse sequence.
    Application of a fat-saturation pre-pulse may also be helpful for discriminating epicardial fat from pathological subepicardial LGE foci.
    Any quantitative approach for LGE quantification was recommended in the LLc, although semi-automated standard deviation (SD)-based thresholding techniques are commonly used today in different clinical settings, primarily in the assessment of acute myocardial infarction (AMI) [28].
    **Limitations:**
    LGE has been extensively validated as a robust and reproducible technique for detecting myocardial fibrosis and myocyte necrosis in patients with AM [26, 29, 30].
    However, LGE detection relies on differences in gadolinium concentration between pathological and “normal” segments. Such differences may not exist if the process is diffuse, or may be insufficient to create contrast in the presence of mild focal involvement.
    Recent data have demonstrated a limited sensitivity of the LGE technique, mainly in patients with less common disease onset such as arrhythmic or cardiomyopathic presentations [31]. These results, correlated with histology, better demonstrate that LGE may be less sensitive for the detection of myocarditis with limited or non-focal myocyte injury.

## Diagnostic performance of LLc

Clinical studies investigating the diagnostic performance of LLc [12] in AM were selected and are reported in Table 2 [12, 16, 21, 30–36].

**Table 2:** Overview of the diagnostic performance of individual conventional CMR criteria and Lake Louise criteria for myocardial characterization in AM; articles are listed in chronological order (see specific references in the text)

First author (reference)	Year	N	EGEr	T2 ratio	LGE	2/3 Positive criteria (LLc)	Reference standard
Abdel-Aty [16]	2005	48	Se 80	Se 84	Se 44	Se 76	Clinical/coronary angiography
			Spec 68	Spec 74	Spec 100	Spec 95	
			Acc 74	Acc 79	Acc 71	Acc 85	
Röttgen [33]	2011	131	Se 49	Se 58	Se 31	/	EMB
			Spec 74	Spec 57	Spec 88		
			Acc 57	Acc 58	Acc 50		
Stensaeth [32]	2012	42	Se 31	Se 57	Se 64	Se 76	Clinical/coronary angiography
			Spec -	Spec -	Spec -	Spec -	
			Acc -	Acc -	Acc -	Acc -	
Lurz [30]	2012	70	Se 76	Se 64	Se 74	Se 81	EMB
			Spe 53	Sp 65	Spec 65	Spec 71	
			Acc 70	Acc 63	Acc 71	Acc 79	
Šramko [36]	2013	42	Se 40	Se 7	Se 87	Se 53	EMB
			Spec 96	Spec 100	Spec 44	Spec 93	
			Acc 76	Acc 66	Acc 60	Acc 78	
Chu [13]	2013	45	Se 66	Se 69	Se 77	Se 80	Clinical/coronary angiography
			Spec 90	Spec 100	Spec 60	Spec 90	
			Acc 72	Acc 76	Acc 73	Acc 82	
Francone [31]	2014	57	Se 61	Se 47	Se 60	Se 61	EMB
			Spec -	Spec -	Spec -	Spec -	
			Acc -	Acc -	Acc -	Acc -	
Radunski [34]	2014	125	Se 63	Se 76	Se 61	Se 84	Clinical/coronary angiography
			Spec 71	Spec 42	Spec 100	Spec 57	
			Acc 59	Acc 70	Acc 67	Acc 79	
Luetkens [21]	2014	66	Se 83	Se 79	Se 75	Se 92	Clinical/coronary angiography
			Spec 42	Spec 61	Spec 100	Spec 80	
			Acc 60	Acc 68	Acc 91	Acc 85	
Pooled data			Se 60	Se 63	Se 59	Se 77	
			Spec 68	Spec 64	Spec 85	Spec 81	
			Acc 63	Acc 63	Acc 71	Acc 78	

*EGEr* early gadolinium enhancement ratio = enhancement_myocardium_/enhancement_skeletal muscle,_ *T2 ratio* = signal intensity_myocardium_/signal intensity_skeletal muscle_, *LGE* late gadolinium enhancement, *LLc* Lake Louise criteria, *Se* sensitivity, *Spec* specificity, *Acc* diagnostic accuracy, *EMB* endomyocardial biopsy

Pooled analysis of results (Table 2) confirmed that LLc (positivity of 2/3 parameters) are more sensitive than each single parameter, and are thus able to partially overcome their diagnostic limitations. Nevertheless, CMR based on LLc yielded suboptimal results: pooled sensitivity of 77 % and specificity of 81 %, slightly lower than the specificity provided by LGE alone (87 %), with diagnostic accuracy of 78 %.

## Prognostic value of CMR

The acute presentation of myocarditis is highly variable, ranging from asymptomatic or mildly symptomatic forms to sudden cardiac death (SCD). AM has been reported to be responsible for at least 5 % of outpatient SCD [37], and this can increase to 42 % in the clinical setting among young patients with AM [38].

The long-term prognosis is usually favorable, but a significant percentage of patients affected by AM experience progressive left ventricle dysfunction and dilatation. Prospective studies have suggested that AM causes at least 10 % of dilated cardiomyopathy [39], and that post-myocarditis scar may represent the substrate of life-threatening ventricular arrhythmias. Therefore, the identification of high-risk patients could have very important clinical implications.

Mahrholdt and colleagues found that left ventricular end-diastolic volume (EDV), the presence of LGE in the interventricular septum, and the total amount of LGE at initial CMR were the strongest independent predictors of impaired ventricular function and ventricular dilatation at follow-up in a cohort of patients with parvovirus B 19 (PVB19) and/or human herpesvirus 6 (HHV6) infections [40]. Moreover, Grün et al. found that LGE was the best independent predictor of both all-cause and cardiac mortality (hazard ratios of 8.4 and 12.8, respectively) in long-term follow-up of 222 consecutive patients with biopsy-proven viral myocarditis [41]. Another interesting result coming from the same cohort of patients was that, independently of left ventricular volumes and function, no patient without LGE experienced SCD [41]. Additionally, Schumm et al. recently reported that a negative CMR in patients referred for clinically suspected myocarditis was associated with good prognosis at follow-up [42].

## Diagnostic role of CMR, based on patient presentation

The wide and expanding use of CMR in the clinical setting of AM is attributable to its unique capacity to comprehensively evaluate the various components of myocardial inflammation cascade.

Significant variations in sensitivity and negative predictive values have been reported in the scientific literature, reflecting both the syndromic nature of the disease and the technical drawbacks of conventional CMR sequences, with evidence that a negative CMR exam cannot reliably rule out a diagnosis of AM.

Diagnostic pathways based on the three main clinical scenarios of AM can be suggested as evidence of the recent literature on the diagnostic accuracy and prognostic value of CMR.

1. *Patients presenting with acute chest pain, positive troponin test results, and typical ST segment abnormalities*
  In the setting of symptomatic patients, for chest pain with typical ST elevation, increased troponin, and preserved LV function, CMR is pivotal in the diagnosis of AM.
  The first role of CMR is the exclusion of alternative diagnoses with similar clinical presentations, such as takotsubo or AMI with non-obstructive coronary artery disease [43–47].
  If the CMR is positive for AM according to LLc, appropriate management needs to be carefully evaluated, taking into account the clinical course and the CMR results, particularly the extent, location, and distribution of LGE [40]. Excellent prognosis is expected in the case of completely normal CMR [42].
  EMB is generally not indicated in this specific clinical scenario, according to the scientific statement regarding the role of EMB in the management of cardiovascular disease published by the American Heart Association (AHA), the American College of Cardiology (ACC), and the European Society of Cardiology (ESC) [48]. However, the position statement of the ESC on myocarditis is not in complete agreement on this point [49].
2. *Patients with new onset of heart failure syndrome of unknown etiology* [16, 43]
  CMR is of value for clarifying the underlying pathophysiology in patients presenting with new-onset heart failure (HF) in the absence of chest pain or known prior myocardial infarction, potentially acting as gatekeeper to invasive coronary angiography (Fig. 3) [50].

**Fig. 3:**
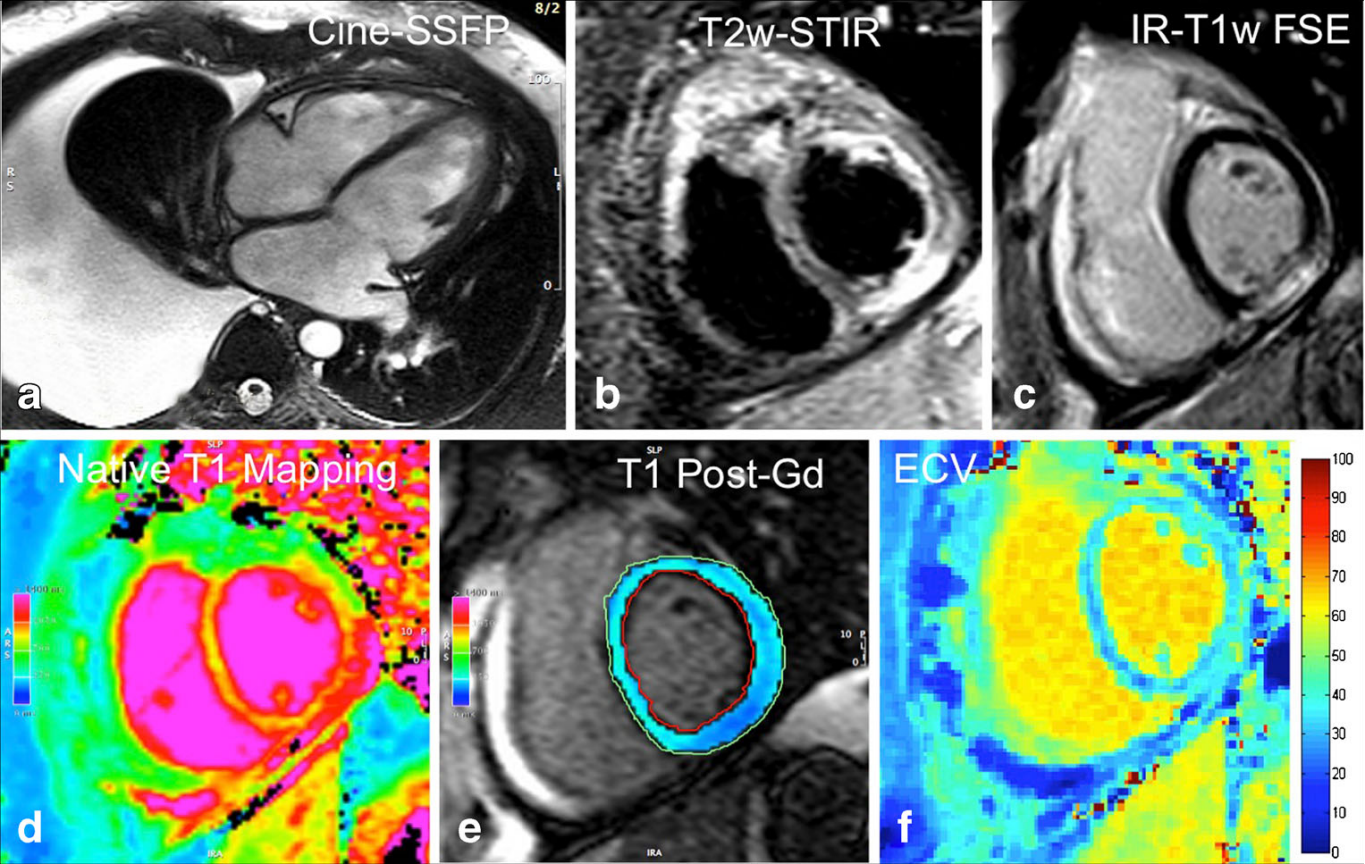
Non-fulminant acute myocarditis in a 44-year-old man presenting with sudden occurrence of signs and symptoms of heart failure (New York Heart Association [NYHA] class III). Cine-SSFP four-chamber end-diastolic frame shows a mildly dilated left ventricular cavity (EDV 187 mL) with significant right-sided pleural effusion; ejection fraction is mildly depressed (44 %). Typical subepicardial striae of high signal intensity are located at the level of the mid-inferior left ventricle on both T2w-STIR (**b**) and LGE images (**c**). Native (**d**) and post-contrast (**e**) T1 mapping scans were also performed using a modified Look-Locker inversion recovery (MOLLI) sequence with a 1.5 T scanner (MAGNETOM Avanto; Siemens Healthcare, Erlangen, Germany), showing abnormally elevated extracellular volume within the mid-inferior segment (36.7 %).
  Post-myocarditis forms, however, are not always recognizable in this specific setting due to a prolonged subacute myocardial inflammation preceding clinical manifestations, and leading to progressive water reabsorption with less evident signal changes [31].
  EMB remains the primary alternative for these patients [51] due to the meaningful impact on subsequent therapeutic decisions [49].
3. *Patients presenting with sudden occurrence of palpitations/lipothymia-hypotension related to the presence of previously unknown ventricular arrhythmias*
  In this clinical scenario, CMR may help to identify AM as the underlying cause of clinical presentation [52], although mild forms of inflammation may result in a negative CMR examination [31].
  CMR plays a role in the identification of other potential causes of previously undiagnosed ventricular arrhythmias, such as arrhythmogenic right ventricular dysplasia, non-compaction disease, atypical forms of hypertrophic cardiomyopathy, and unknown chronic myocardial scars [52]. CMR can also detect unexpected causes of acute myocardial injury such as an unrecognized myocardial infarction. Moreover, CMR results may provide important prognostic information that should be considered in patient management.
  Indication of EMB is not well established in these patients: EMB should be considered only in exceptional cases of unexplained ventricular arrhythmias [51], but it is generally recommended when clinical and imaging evaluation provides significant evidence of AM [49].

## New insights and future developments: myocardial mapping

The major limitations of current LLc are the qualitative (LGE depiction) or semiquantitative (T2 ratio and EGEr) nature of the diagnostic criteria, which significantly affects the sensitivity of the CMR technique in the characterization of global myocardial processes.

To overcome many of these limitations, current research has developed absolute quantitative approaches such as T1 and T2 mapping techniques.

### Native T1 mapping

Measurement of native myocardial longitudinal relaxation time (T1 mapping) has emerged as a novel quantitative approach to enable the depiction of diffuse inflammatory processes. It is based on the generation of parametric maps representing the T_1_ relaxation times encoded in each pixel of any region of the heart, without the need to normalize measurements to a reference standard tissue [14, 53, 54].

The basic principle of this technique relies on the concept that pixel signal intensity depends on the relaxation of hydrogen nuclei protons in a static magnetic field, which varies in the presence of oedema, fat infiltration, and fibrosis [21].

Specifically, the prolongation of T_1_ relaxation time in AM is related to an increase in both total and relative water content in the intra- and extracellular space due to tissue oedema and expansion of intercellular space [55]. A second phenomenon, further enhancing T1 prolongation, is the altered electrolyte distribution in the inflamed myocardial tissue, affecting the motion of protons [56]. An initial experience published by Ferreira et al. showed excellent diagnostic performance of native T1 mapping in the clinical setting of AM, with around 90 % overall sensitivity, specificity, and diagnostic accuracy [57].

More recently, Luetkens et al. convincingly demonstrated the importance of a CMR multiparametric approach [21]. Native myocardial T1 mapping proved to be superior to the EGEr technique and, alone, provided sensitivity comparable to that obtained with the established LLc (92 vs. 92 %, respectively) [19, 21]. Hinojar et al. also reported the applicability of a new diagnostic algorithm using native T1 for differentiating acute from convalescent myocarditis (native T1 values: 940 vs. 1064 vs. 995 ms at 1.5 T and 1045 vs. 1189 vs. 1099 ms at 3 T in controls, acute myocarditis, and convalescent myocarditis, respectively) [35].

Notably, a marked variation in diagnostic thresholds among normal subjects has been reported in the literature [58]. This variation in normal values of myocardial T1 is a reflection of its direct dependence on magnetic field strength, different physiologic parameters (mainly age, gender, and heart rate), scanning parameters (mainly flip angle, matrix, and slice thickness), and image analysis methods. One of the major risks is the partial volume effect arising from over-inclusion of neighbouring tissue [59]. Hence, in agreement with current consensus statement [53], the validation of the imaging protocol in each site using a reference sample of “normal subjects” is highly recommended in order to minimize measurement errors. A more robust application of this approach would certainly require greater standardization and larger validation studies, which are currently lacking in the literature.

### Pre- and post-contrast T1 mapping with extracellular volume fraction

Extracellular volume fraction (ECV) provides a direct measurement of the size of the extracellular space. ECV map generation is based on a pixel-wise co-registration and comparison of native and post-contrast T1 maps, adjusted for the patient’s hematocrit [53]. In AM, an increase in ECV reflects myocardial oedema and inflammation as well as myocyte necrosis and subsequent myocardial fibrosis [21, 34, 60, 61]. In recent studies, ECV alone achieved diagnostic accuracy of approximately 75 % [21, 34, 36], which can improve to 90 % if ECV ≥ 27 % is combined with the presence of LGE in a stepwise diagnostic approach [34].

### T2 mapping

An increased signal in T2-weighted images, which is linked to T_2_ relaxation time (T2-rt) lengthening, is a well-known MR sign of cardiac and skeletal muscle damage, reflecting heterogeneous phenomena such as oedema and inflammation. Qualitative assessment of T2-weighted imaging is limited by signal intensity heterogeneities and by the absence of the “perfect” reference tissue for signal normalization. Direct measurement of T2-rt accurately reflects inflammation in the skeletal muscles [62] and also allows non-invasive monitoring of inflammatory infiltration in the same setting [63]. Hence, T2 mapping also holds promise as an attractive alternative for the assessment of myocardial inflammation, particularly in the case of diffuse oedema rather than focal involvement.

A T2-prepared steady-state free precession (T2p-SSFP) technique has been described as a means of minimizing the problems associated with current cardiac T2W imaging methods, and of providing an accurate assessment of myocardial oedema [64]. This T2p-SSFP approach allows the generation of three T2w images, each with different T2 preparation times, in a single breath-hold (seven R–R intervals required). Images obtained with this technique are less sensitive than those of classical T2w-STIR to motion artefacts induced by arrhythmias or imperfect breath-hold, and can be processed to fit the T2 decay curve at each pixel to yield a T2 map. These T2 maps also solve the critical problem of signal heterogeneity linked to the use of multi-element surface coils that typically affects STIR images. Results of T2 mapping have shown promise in the detection of myocardial involvement in acute inflammatory cardiomyopathy, in particular in patients with myocarditis and takotsubo cardiomyopathy, where T2 mapping allows the delineation of a greater extent of myocardial involvement than wall motion abnormalities, T2w-STIR, or LGE [65]. However, no other studies have confirmed the value of myocardial T2 mapping in AM, and thus further evidence from multicenter trials is needed to prove its reproducibility and to assess its potential clinical role in patients with suspected AM.

## Conclusions

**Fig. 4:**
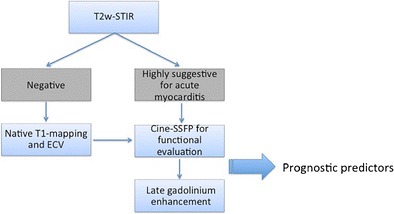
Revised diagnostic algorithm for the clinical workup in patients with clinically suspected acute myocarditis. Routine inclusion of T1 mapping techniques (native and ECV) in the scanning protocol would enable the coupling of the high specificity of T2-STIR and LGE techniques with the increased sensitivity of T1-relaxation changes measurements (particularly in mild focal or diffuse forms of disease). According to the literature, a combination of functional data and inflammation/necrosis imaging correlates provided by CMR may serve as a predictor of functional and clinical recovery at follow-up (see text for further explanation).

Since the publication of the JACC White Paper entitled “Cardiovascular Magnetic Resonance in Myocarditis” in 2009, the relevance of CMR as a component of the clinical workup in AM has increased dramatically. Several years of application of LLc have allowed for a better understanding of their value and limits, revealing flaws in providing wishful diagnostic results in some clinical situations. The EGE seems to be the weakest point of LLc, both with respect to the presence of ambiguity and, more importantly, with the strong artefacts that very frequently affect this technique. T2w-STIR sequences, although potentially affected by artefacts in some patients and limited in the assessment of mild and diffuse forms of myocardial involvement, may provide an intuitive and rapid means of ascertaining active inflammation in focal forms of disease. LGE is primarily affected by low sensitivity, but has significant value in differential diagnosis and prognostic evaluation. New perspectives in the diagnostic potential of CMR were highlighted by the recent introduction of mapping sequences, which provide insight into quantitative assessment of inflammatory myocardial changes. New scientific evidence supports the value and robustness of T1 mapping techniques in the diagnosis of AM, with the chief advantage of increasing CMR sensitivity. However, larger multicenter trials are needed to develop a standardized T1/T2 mapping technique and to define widely accepted normal thresholds values. On the other hand, a step-by-step simple CMR protocol including T2w-STIR, T1 mapping, and LGE (Fig. 4) could be hypothesized and tested experimentally and in clinical practice.

## Abbreviations

AM: Acute myocarditis
AMI: Acute myocardial infarction
ARVD/C: Arrhythmogenic right ventricular dysplasia/cardiomyopathy
ceSSFP: Contrast-enhanced steady-state free precession
CMP: Cardiomyopathy
CMR: Cardiovascular magnetic resonance
ECV: Extracellular volume fraction
EDV: End-diastolic volume
EGEr: Early gadolinium enhancement ratio
EMB: Endomyocardial biopsy
Gd-BOPTA: Gadolinium benzyloxy-propionic-tetraacetic acid
Gd-DTPA: Gadopentetate dimeglumine
FSE: Fast spin echo
HHV6: Human herpesvirus 6
IR-GRE: Inversion recovery gradient echo
IR-SSFP: Inversion recovery steady-state free precession
LGE: Late gadolinium enhancement
LLc: Lake Louise criteria
LV: Left ventricle
NT-pro BNP: N-terminal of the prohormone brain natriuretic peptide
PVB19: Parvovirus B 19
PSIR: Phase-sensitive inversion recovery
ROI: Region of interest
SCD: Sudden cardiac death
SSFP: Steady-state free precession
TSE: Turbo spin echo
TT-CMP: Takotsubo cardiomyopathy
T2p-SSFP: T2-prepared steady-state free precession
T2w-STIR: Short-tau inversion recovery prepared fast spin echo
